# Unveiling the rhizosphere microbiome of *Dendrobium*: mechanisms, microbial interactions, and implications for sustainable agriculture

**DOI:** 10.3389/fmicb.2025.1531900

**Published:** 2025-01-29

**Authors:** Surendra Sarsaiya, Archana Jain, Ranjan Singh, Qihai Gong, Qin Wu, Jishuang Chen, Jingshan Shi

**Affiliations:** ^1^Key Laboratory of Basic Pharmacology and Joint International Research Laboratory of Ethnomedicine of Ministry of Education, Zunyi Medical University, Zunyi, China; ^2^Bioresource Institute for Healthy Utilization, Zunyi Medical University, Zunyi, China; ^3^Department of Microbiology, Faculty of Science, Dr. Rammanohar Lohia Avadh University, Ayodhya, India

**Keywords:** *Dendrobium*, rhizosphere microbiome, plant-microbe interactions, microbiome engineering, sustainable agriculture

## Abstract

The rhizosphere microbiome plays a critical role in plant health and productivity by fostering beneficial microbial interactions that support nutrient cycling, stress tolerance, and disease suppression. In the context of *Dendrobium*, understanding its interactions is essential for optimizing cultivation and promoting sustainable agricultural practices. This review explores the rhizosphere microbiome of *Dendrobium*, focusing on the mechanisms and microbial interactions that contribute to plant health, stress tolerance, and growth and their implications for sustainable agriculture. This study highlights the diverse composition of microbial communities in the *Dendrobium* rhizosphere, including key bacteria (e.g., *Pseudomonas fluorescens* and *Bacillus subtilis*), fungi (e.g., *Glomus* spp.), and biocontrol agents (*Trichoderma* spp.), and discusses their roles in nutrient cycling, disease suppression, and plant growth promotion. This review emphasizes the significance of plant-microbe signaling, such as the production of flavonoids, phytohormones, and strigolactones, in shaping the microbial environment and enhancing plant resilience. Additionally, it addresses modern techniques for analyzing microbial communities, including metagenomics and next-generation sequencing, and their applications in advancing precision agriculture. Future research should focus on bridging knowledge gaps related to genotype-microbiome interactions, exploring emerging microbial consortia and enhancing the integration of microbiome management in precision agriculture systems to improve plant health and productivity.

## 1 Introduction

The rhizosphere microbiome plays a crucial role in enhancing the health, resilience, and growth of *Dendrobium* plants, a genus of orchids valued for their medicinal properties and ornamental appeal (Zhu et al., 2024). This complex community of microorganisms surrounding plant roots significantly influences plant health by extending the functional capabilities of the plant, often referred to as its “second genome” (Xie et al., 2024). The diversity of microbial species in the rhizosphere is immense, with tens of thousands of species interacting with plant roots, which can lead to improved plant growth and resilience against environmental stressors (Chen L. et al., 2023). One of the primary mechanisms by which the rhizosphere microbiome enhances plant health is the action of plant growth-promoting rhizobacteria (PGPRs). These beneficial bacteria can colonize the roots and promote growth by facilitating nutrient acquisition and stimulating plant hormones (Zhao R. et al., 2023; Saikia and Thakur, 2024).

In *Dendrobium* plants, the presence of PGPRs can lead to enhanced nutrient uptake, particularly of essential elements such as nitrogen and phosphorus, which are critical for growth (Bloch and Ghosh, 2024). Additionally, PGPRs can produce metabolites such as indole acetic acid (IAA) and gibberellins, which directly promote plant growth (de Medeiros et al., 2023). Moreover, the rhizosphere microbiome contributes to biocontrol mechanisms that suppress plant pathogens, thereby enhancing the resilience of *Dendrobium* plants to diseases (Xie et al., 2024). This is particularly important in the context of sustainable agriculture, in which reducing reliance on chemical pesticides is a growing concern. The interactions among various microbial species in the rhizosphere can create a balanced ecosystem that supports plant health by outcompeting harmful pathogens and promoting beneficial microbial activity (Zhao R. et al., 2023). The ability of *Dendrobium* plants to shape their rhizosphere microbiomes further underscores the dynamic relationship between plants and their microbial communities. Different *Dendrobium* species can host specific microbial communities, which can be tailored to enhance their growth and resilience in varying environmental conditions (Yuan et al., 2024). This adaptability is vital for the conservation of *Dendrobium* species, many of which are threatened by overcollection and habitat loss. Moreover, the production of secondary metabolites, such as dendrobine, is enhanced by the presence of specific microbial communities, indicating that these interactions are not merely supportive but also integral to the plant’s biochemical processes (Sarsaiya et al., 2024). The structure of these microbial communities can vary significantly based on environmental conditions and specific plant organ types, further emphasizing the need for a diverse microbial ecosystem to meet the specialized requirements of *Dendrobium*.

Understanding the dynamics of root exudation and its effect on microbial interactions is essential for optimizing the health and growth of *Dendrobium* plants. Despite the promising benefits of the rhizosphere microbiome, several challenges remain. The vast majority of rhizosphere microorganisms are unculturable (Saikia and Thakur, 2024), limiting our understanding of their roles and interactions (Xie et al., 2024). Additionally, the domestication of *Dendrobium* and other plants has led to a reduction in genetic diversity, which may affect their ability to establish beneficial associations with rhizosphere microbes (Chen L. et al., 2023). Although the rhizosphere microbiome offers significant potential for enhancing the health and resilience of *Dendrobium* plants, further research is needed to unravel the complexities of microbial interactions and their implications for sustainable cultivation practices. Addressing these challenges is crucial for harnessing the full potential of the rhizosphere microbiome in agricultural biotechnology. The objective of this review article was to provide a comprehensive overview of the rhizosphere microbiome of *Dendrobium* orchids, with a particular focus on the mechanisms and microbial interactions that contribute to plant health and growth. It explores the role of key microbial communities, including bacteria, fungi, and their signaling pathways, in shaping the root environment, enhancing nutrient uptake, suppressing pathogens, and promoting stress resilience. This review also highlights modern techniques for studying the microbiome, the potential of engineered microbiomes for sustainable agriculture, and the integration of microbiome research with precision farming practices. Ultimately, this study aimed to bridge knowledge gaps and identify emerging research areas for optimizing *Dendrobium* cultivation and promoting its ecological and agricultural significance.

## 2 The rhizosphere microbiome: composition and diversity

The *Dendrobium* rhizosphere microbiome is characterized by a complex and diverse community of microorganisms that play a crucial role in plant health and nutrient uptake. This microbiome is significantly influenced by the physical and chemical properties of the rhizosphere, which are, in turn, affected by the genetics of the host plant (Li et al., 2021a; Singh et al., 2023). The composition of the *Dendrobium* rhizosphere microbiome is dominated by several bacterial phyla, including Proteobacteria, Actinobacteria, and Bacteroidetes, which are essential for various ecological functions (Wang H. et al., 2022; Li X. et al., 2024). High-throughput sequencing technologies have facilitated the detailed characterization of this microbial diversity, allowing researchers to identify 11,179 operational taxonomic units (OTUs) within the *Dendrobium* rhizosphere (Zuo et al., 2021). This resolution is critical for understanding the intricate relationships between microbial communities and their plant hosts. Mycorrhizal fungi, including those from the genus *Glomus*, were particularly significant in the rhizosphere of *Dendrobium*. They form arbuscules within the roots, facilitating efficient nutrient absorption (Favre-Godal et al., 2021). The presence of mycorrhizal fungi not only improves nutrient uptake but also enhances the resilience of plants against environmental stresses and pathogens. In addition to fungi, various bacterial species contribute to the health of the *Dendrobium* rhizosphere. *Pseudomonas fluorescens*, a Gram-negative bacterium, is commonly found in agricultural soils and is known for its ability to protect plants from pathogens while promoting growth (Pavlova et al., 2017; Li Z. et al., 2024). Similarly, *Bacillus subtilis* is recognized for its plant growth-promoting properties and its role in suppressing plant pathogens, making it another important resident of the rhizosphere (Sun et al., 2023). Nitrogen-fixing bacteria such as *Bradyrhizobium denitrificans* and members of the *Rhizobium* genus are also present in the rhizosphere, contributing to nitrogen availability, which is vital for plant growth (Tshikhudo et al., 2023). These bacteria form symbiotic relationships with legumes, but their presence in the rhizosphere of *Dendrobium* spp. can enhance the overall nutrient profile of the soil. Moreover, *Trichoderma* spp., which are known for their biocontrol properties against plant pathogens, are frequently found in the rhizosphere and contribute to the promotion of plant growth (Sarsaiya et al., 2020; Chua et al., 2023). Actinobacteria, another group of beneficial bacteria, play a significant role in soil health and nutrient cycling, further supporting the growth of *Dendrobium* (Zhu et al., 2024).

Microbial community structure varies significantly among different *Dendrobium* species, indicating that specific plant traits can drive the selection and activities of associated microorganisms (Chen L. et al., 2023; Zhao R. et al., 2023; Chen W. et al., 2023). The interactions between plants and their microbiomes are vital for enhancing plant growth and health as these relationships can influence nutrient uptake and overall plant performance (Zhu et al., 2024; Xie et al., 2024). Furthermore, environmental factors such as nitrogen, phosphorus, and pH play a significant role in shaping the composition of rhizosphere microbial communities, underscoring the importance of soil conditions in microbial ecology (Xie et al., 2024; Li Y. et al., 2024). The vast array of microbial life found in the rhizosphere can be 10 to 1,000 times more abundant than in bulk soil, emphasizing the critical role of this niche in supporting plant health and productivity (Zuo et al., 2021). Understanding the dynamics of the *Dendrobium* rhizosphere microbiome is essential for harnessing its potential in agricultural practices and conservation. As research continues to uncover the complexities of these microbial communities, it becomes increasingly clear that the rhizosphere serves as a vital interface for resource exchange between plants and their soil environment, ultimately influencing plant health and ecosystem sustainability (Chauhan and Attri, 2024).

**Figure 1:**
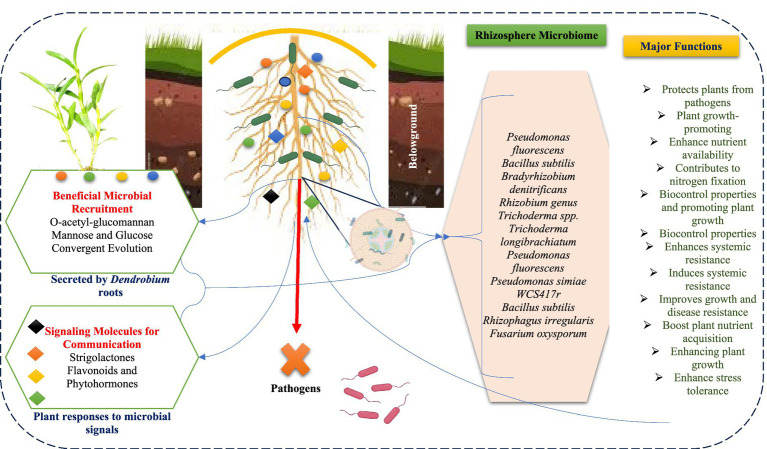
Overview of microbial diversity and beneficial interactions in the *Dendrobium* rhizosphere.

The composition of the *Dendrobium* rhizosphere microbiome is influenced by several interrelated factors, including root age, nitrogen addition, mycorrhizal status, plant genotype, and immune responses (Chen L. et al., 2023; Yuan et al., 2024; Wang S. S. et al., 2022). Root age plays a significant role, as bacterial activity in the rhizosphere increases over time, indicating that older roots contribute to a more diverse microbial community due to the carbon-rich exudates they release (Bais et al., 2006; Bhutia et al., 2023). Additionally, the source of nitrogen added to the soil can affect bacterial activity, with variations in pH influencing the composition of microbiome (Zhu et al., 2024). Mycorrhizal status, particularly arbuscular mycorrhizal colonization, also impacts the rhizosphere bacteria differently across plant species, suggesting that the interaction between mycorrhizae and specific plant types is crucial for microbiome dynamics (Herrera et al., 2022; Yu et al., 2022). Furthermore, plant genotype is a critical factor, as different genotypes can selectively promote or suppress specific bacterial and fungal populations through their root exudates, thereby shaping the microbiome (Raj et al., 2024a; Liu et al., 2024). Finally, the immune response of plants, especially through PAMP-triggered immunity, significantly alters the microbiome composition, indicating that the defense mechanisms of plants can influence microbial community structure (Shelake et al., 2019). Collectively, these factors highlight the complex interplay between plant characteristics and microbial communities in the rhizosphere, underscoring the importance of understanding these dynamics to optimize plant health and productivity (Table 1 and Figure 1).

**Table 1:** Rhizosphere microbial communities and their roles in *Dendrobium* plant health and growth.

Aspect	Details	References
Bacterial phyla in *Dendrobium* rhizosphere		
Proteobacteria	Essential for nutrient cycling and pathogen suppression	Li X. et al. (2024)
Actinobacteria	Play a significant role in soil health and nutrient cycling	Zhu et al. (2024)
Bacteroidetes	Important for nutrient degradation and transformation	Li et al. (2024)
Key beneficial bacteria		
*Pseudomonas fluorescens*	Protects plants from pathogens while promoting growth	Li X. et al. (2024)
*Bacillus subtilis*	Plant growth-promoting bacterium that suppresses plant pathogens	Sun et al. (2023)
*Bradyrhizobium denitrificans*	Nitrogen-fixing bacteria that enhance nutrient availability	Tshikhudo et al. (2023)
*Rhizobium* genus	Contributes to nitrogen fixation, improving nutrient profile in the rhizosphere	Tshikhudo et al. (2023)
Mycorrhizal fungi		
*Glomus* spp.	Facilitate nutrient uptake, especially phosphorus, and enhance stress tolerance	Favre-Godal et al. (2021)
Biocontrol fungi		
*Trichoderma* spp.	Known for biocontrol properties against plant pathogens and promoting plant growth	Chua et al. (2023)
*Trichoderma longibrachiatum*	Known for biocontrol properties against plant pathogenic bacteria	Sarsaiya et al. (2020)
Microbial community structure		
Plant traits shaping microbial communities	Specific plant traits of *Dendrobium* species shape the selection and activities of associated microorganisms	Chen L. et al. (2023)
Microbial variation	Variations in microbial communities driven by plant genotype, root exudates, and environmental factors	Xie et al. (2024) and Li Y. et al. (2024)
Plant growth-promoting rhizobacteria (PGPR)		
*Pseudomonas fluorescens*	Enhances systemic resistance and promotes plant health	Ye et al. (2019)
*Pseudomonas simiae* WCS417r	Induces systemic resistance against pathogens	Ye et al. (2019)
*Bacillus subtilis*	Improves growth and disease resistance	Chen X. et al. (2023)
Arbuscular mycorrhizal fungi (AMF)		
*Rhizophagus irregularis*	Forms symbiotic relationships to boost plant nutrient acquisition	Wang et al. (2018)
Endophytic microbes		
*Trichoderma* spp.	Provides protection against pathogens and improves stress tolerance	Meng et al. (2024)
*Fusarium oxysporum*	Symbiotic endophyte enhancing plant growth	Zhu et al. (2022)
Microbial communities and metabolic pathways		
Actinobacteria	Contributes to plant health by synthesizing secondary metabolites, enhancing growth	Pavlova et al. (2017)
Flavonoid-producing microbes	Support microbial community composition in *Dendrobium* root	Favre-Godal et al. (2020)
Beneficial microbial recruitment		
O-acetyl-glucomannan	Polysaccharide secreted by *Dendrobium* roots to recruit beneficial microbes	He et al. (2017)
Mannose and glucose	Monosaccharides from *Dendrobium* roots enhance microbial recruitment	Pham et al. (2023)
Convergent evolution	Genotype-microbiome interactions result in similar adaptive traits across different environmental niches, optimizing growth and disease resistance	Seem et al. (2024) and Yuan et al. (2020)
Signaling molecules in microbe-plant communication		
Strigolactones	Signaling molecules that enhance mycorrhizal interactions	Wang et al. (2024)
Flavonoids and phytohormones	Crucial signaling molecules for plant responses to microbial signals	Wang Y. et al. (2022)

## 3 Available modern techniques for studying the rhizosphere microbiome

The study of the *Dendrobium* rhizosphere microbiome employs several advanced molecular techniques that enhance our understanding of microbial diversity and functionality. Key among these techniques are metagenomics, next-generation sequencing (NGS), and 16S rRNA sequencing (Herrera et al., 2022; Tamošiūnė et al., 2019). Metagenomics allows researchers to analyze genetic material directly from environmental samples, providing insights into the complex interactions within the *Dendrobium* rhizosphere (Chen L. et al., 2023). This approach is crucial for understanding the diversity of microbial communities as it enables the identification of both culturable and non-culturable microbes present in the soil (Jamwal et al., 2023). By utilizing NGS platforms, researchers can perform high-throughput sequencing of DNA, which facilitates detailed analysis of bacterial communities and their functional roles in the rhizosphere (Wang H. et al., 2022; Li S. et al., 2024). 16S rRNA sequencing is another pivotal method that focuses on amplifying and sequencing the 16S ribosomal RNA gene, which is essential for identifying and comparing bacterial species within a sample (Zuo et al., 2021). This technique is particularly valuable for profiling the diversity of microbial communities as it provides a reliable means of distinguishing between different bacterial taxa. Despite the advantages of these techniques, they have limitations. For instance, although metagenomics offers a comprehensive view of microbial diversity, it can be biased toward certain taxa because of the uneven distribution of DNA in environmental samples. Additionally, the complexity of data generated from NGS can pose challenges in data interpretation and analysis, requiring sophisticated bioinformatics tools and expertise (Adeleke et al., 2023). Moreover, 16S rRNA sequencing, which is effective for identifying bacterial communities, does not provide insights into the functional capabilities of these microbes. This limitation can be addressed by integrating metatranscriptomics, which analyzes RNA to assess gene expression and metabolic activity within the microbial community (Chen L. et al., 2023; Zuo et al., 2021). However, metatranscriptomics also faces challenges, such as the instability of RNA and the need for immediate processing of samples to prevent degradation.

Various methods, including metagenomics, direct soil-phospholipid fatty acid (PLFA), colony-forming unit (CFU)-PLFA, and community-level physiological profiling (CLPP) (BIOLOG), each provide unique insights into microbial community composition and activity, which are crucial for understanding their ecological roles (Söderberg et al., 2004). Metagenomics, for instance, allows for the comprehensive analysis of total DNA from microbial communities, revealing genetic diversity and potential functional capabilities within the rhizosphere (Wang H. et al., 2022). This technique is particularly powerful because it captures the genetic material of non-cultivable microbes, which traditional methods often overlook. By employing metagenomic sequencing, researchers can gain insights into the functional potential of microbial communities, thereby enhancing the accuracy of functional predictions (Jain et al., 2024). In addition to metagenomics, the incorporation of thymidine and leucine is vital for measuring the bacterial activity in the rhizosphere. These methods have demonstrated that bacterial activity can be significantly higher in the rhizosphere than bulk soil, which is essential for understanding the dynamics of microbial interactions with plant roots (Söderberg and Bååth, 2004; Cottrell and David, 2003). The direct measurement of bacterial activity using these techniques provides a more nuanced view of how microbial communities respond to environmental changes and plant influences. Furthermore, profiling microbial community composition using CLPP (BIOLOG) and PLFA methods (both direct soil-PLFA and CFU-PLFA) contributes to functional predictions by elucidating the community structure (Velasco et al., 2010). These techniques allow researchers to assess how different plant species affect the microbial community, which is crucial for understanding ecological interactions in the rhizosphere. The findings indicate that plant species have a more pronounced effect on bacterial communities than arbuscular mycorrhizal (AM) colonization, highlighting the importance of plant-microbe interactions in shaping microbial dynamics (Xie et al., 2024; Chen L. et al., 2023). Ultimately, the integration of these microbiome profiling techniques enhances the ability of the *Dendrobium* rhizosphere microbiome to make accurate functional predictions. By combining genetic, compositional, and activity-based data, researchers can develop a more comprehensive understanding of the metabolic and ecological roles of microbial communities, leading to improved insights into plant health and growth (Chen L. et al., 2023; Zhao R. et al., 2023). This multifaceted approach is essential for advancing our knowledge of plant-microbe interactions and optimizing the health of *Dendrobium* plants (Table 2).

**Table 2:** Modern techniques for analyzing rhizosphere microbial communities.

Technique	Description	Advantages	Limitations	References
Metagenomics	Analysis of total genetic material from environmental samples	Identifies culturable and non-culturable microbes; reveals genetic diversity and potential functions	Biased toward certain taxa; requires advanced bioinformatics tools	Chen L. et al. (2023) and Jamwal et al. (2023)
Next-generation sequencing	High-throughput DNA sequencing for detailed analysis of bacterial communities and functions	High-resolution data; suitable for large-scale studies	Complexity of data interpretation; expensive and requires expertise	Wang H. et al. (2022) and Li X. et al. (2024)
16S rRNA sequencing	Amplifies and sequences 16S ribosomal RNA gene to identify and compare bacterial species	Reliable profiling of microbial diversity; identifies bacterial taxa	Limited functional insights; focuses only on bacterial composition	Zuo et al. (2021)
Metatranscriptomics	Analysis of RNA to assess gene expression and microbial metabolic activity	Provides insights into the functional activities of microbes	RNA instability; requires immediate processing of samples	Chen L. et al. (2023) and Zuo et al. (2022)
Direct soil-PLFA	Phospholipid fatty acid analysis to profile microbial community composition directly from soil	Provides insight into community structure; suitable for linking structure to function	May not capture all microbial activity	Söderberg et al. (2004)
CFU-PLFA	Combines culture-based methods with PLFA for profiling microbial communities	Links culturable microbes to community composition; provides functional insights	Focuses on culturable microbes; excludes non-culturable microbes	Söderberg et al. (2004)
CLPP (BIOLOG)	Community-level physiological profiling to assess microbial community function based on substrate utilization	Evaluates functional diversity; reveals how plant species influence microbial communities	The limited scope of substrates; may not reflect actual *in situ* microbial activity	Velasco et al. (2010)
Thymidine/leucine incorporation	Measures bacterial activity in the rhizosphere by quantifying DNA and protein synthesis	Direct measurement of bacterial activity; sensitive to environmental and plant-root interactions	Focused on bacterial activity only; does not provide community composition data	Söderberg and Bååth (2004) and Cottrell and David (2003)

## 4 Mechanisms of microbiome contribution to *Dendrobium* health

The rhizosphere microbiome plays a crucial role in enhancing the health of *Dendrobium* through various mechanisms that facilitate the nutrient acquisition, provide defense against pathogens, and promote overall plant resilience. One of the primary functions of the rhizosphere microbiome is to enhance nutrient uptake, which is vital to the growth and health of *Dendrobium* spp. This is achieved through the interaction of plants with beneficial microorganisms that can improve the availability of essential nutrients in the soil (Zhu et al., 2024; Chen L. et al., 2023; Zuo et al., 2021). In addition to nutrient acquisition, the rhizosphere microbiome significantly contributes to defense against pests and diseases. Microorganisms in the rhizosphere can suppress harmful pathogens, thereby reducing disease incidence and promoting plant health (Saikia and Thakur, 2024; Sarsaiya et al., 2020). This pathogen suppression is often mediated by specific groups of bacteria known as plant growth-promoting rhizobacteria (PGPR), which not only colonize the roots but also enhance plant growth and health through various mechanisms, including the production of growth-promoting hormones (Favre-Godal et al., 2021; Saikia et al., 2022). Moreover, the rhizosphere microbiome aids *Dendrobium* in coping with abiotic stresses, such as drought and salinity, which are critical for maintaining plant resilience in changing environmental conditions (Singh et al., 2023; Pandey et al., 2023). The ability of these microorganisms to stimulate plant hormone production further supports the adaptive responses of plants to stress (Xie et al., 2024; Wang H. et al., 2022). The interactions between *Dendrobium* and its associated microbes are complex and involve various signaling mechanisms that enhance nutrient uptake and disease resistance (Zhu et al., 2024; Teixeira da Silva et al., 2015b). For instance, microbial antagonism, where certain rhizosphere microorganisms act against pathogens, is a vital aspect of this protective mechanism (Xie et al., 2024; Saikia and Thakur, 2024; Shen et al., 2020).

Key cycling pathways facilitated by microbial interactions in the rhizosphere include nitrogen fixation, phosphorus solubilization, and mobilization of essential nutrients. These processes are vital for enhancing nutrient availability, which directly influences the health and productivity of *Dendrobium* orchids (Alibrandi et al., 2021; Martínez-Viveros et al., 2010; Kour et al., 2019). Microbial communities in the rhizosphere, particularly those composed of plant growth-promoting rhizobacteria (PGPR) and mycorrhizal fungi, are instrumental in nutrient cycling. PGPR enhance nutrient availability by suppressing pathogens and promoting plant growth through various mechanisms, including the production of growth-promoting substances (Ayaz et al., 2023; Rajendran et al., 2023; Ren et al., 2023). Mycorrhizal fungi form symbiotic relationships with *Dendrobium* roots, aiding in nutrient absorption, particularly phosphorus, which is essential for plant growth and development (Chakraborty et al., 2023; Goh et al., 2024). These fungi enhance the mobilization of nutrients from organic substrates, thereby improving the overall nutrient uptake efficiency of the plants (Dutta et al., 2024; Raj et al., 2024b). The rhizosphere microbiome is a dynamic environment where diverse bacterial communities, such as those from the phyla Proteobacteria, Actinobacteria, and Bacteroidetes, interact with plant roots to influence nutrient cycling (Zhu et al., 2024; Chen L. et al., 2023). These interactions facilitate various nutrient cycling pathways, including nitrogen fixation, which enriches the soil with bioavailable nitrogen, and phosphorus solubilization, which makes this critical nutrient more accessible to plants (Xie et al., 2024; Tian et al., 2024). The presence of beneficial microbes in the rhizosphere not only enhances nutrient availability but also contributes to the overall health of the plant by improving resistance to diseases and environmental stresses (Zhu et al., 2024; Yu J. B. et al., 2024). Additionally, the root exudates released by *Dendrobium* orchids play a significant role in shaping the microbial community structure in the rhizosphere. These exudates can attract beneficial microbes and stimulate microbial activity, further enhancing nutrient cycling processes (Li Y. et al., 2024; Li S. et al., 2024). Flavonoids, as secondary metabolites, mediate plant-microbe interactions, influencing the composition of the root microbiome and enhancing nutrient uptake (Chen L. et al., 2023; Yu J. B. et al., 2024).

The manipulation of the microbiome presents a promising strategy to enhance the tolerance and resilience of *Dendrobium* orchids to both biotic and abiotic stresses (Martínez-Chávez et al., 2024). *Dendrobium* orchids, known for their medicinal properties and resilience to environmental challenges, can significantly benefit from the interactions with beneficial microorganisms, particularly through the use of plant growth-promoting rhizobacteria (PGPR) and endophytes (Zhao Y. et al., 2023). Microbiome manipulation involves altering the microbial communities associated with plants to improve their growth and stress tolerance (Rai et al., 2023). This approach is particularly relevant for *Dendrobium* orchids, as these plants have established symbiotic relationships with various microorganisms, including mycorrhizal fungi and endophytes, which enhance nutrient uptake and stress resilience (Pandey et al., 2023; Mohan and Joshi, 2024). For instance, the presence of specific endophytic fungi has been shown to promote growth and improve the overall health of *Dendrobium* species, indicating that these microorganisms play a crucial role in the ability of orchids to withstand stress (Chen W. et al., 2023; Yu C. et al., 2024). Furthermore, the resilience of *Dendrobium* orchids to biotic stressors such as pests and diseases can be enhanced through microbiome manipulation. The beneficial microorganisms can help in developing biotic stress tolerance by improving the defense mechanisms of plants against pathogens (Liu et al., 2024; Yu J. B. et al., 2024). This is particularly important, as *Dendrobium* orchids face various biological threats in their natural habitats, and enhancing their innate resistance through microbial interactions could lead to more sustainable cultivation practices. In addition to biotic stress, the resilience of *Dendrobium* orchids to abiotic stressors, such as drought and salinity, can also be improved through microbial interactions (Singh et al., 2023; Han et al., 2024). The beneficial bacteria and fungi associated with the roots can enhance the ability of plants to absorb water and nutrients, thereby improving their overall stress tolerance (Saikia and Thakur, 2024; Goh et al., 2024). This is particularly relevant in the context of climate change, in which environmental stress is becoming increasingly prevalent.

In addition, the microbiome contributes to disease resistance through various biochemical pathways. Endophytic fungi associated with *Dendrobium* have been shown to enhance growth and increase chemical content, which, in turn, improves the orchids’ resistance to pathogens (Chen L. et al., 2023; Liu et al., 2023). Certain endophytic bacteria also exhibit antimicrobial properties that can suppress harmful pathogens, further promoting plant health (de Medeiros et al., 2023; Ayaz et al., 2023). This antimicrobial activity is crucial, as it helps creating a protective barrier against diseases that could otherwise compromise the vitality of the plants. Moreover, the diversity of the microbial communities associated with *Dendrobium* is essential for its health. A rich microbial diversity can lead to a more robust defense system, allowing the plant to better cope with environmental stressors and pathogens (Pandey et al., 2023; Gowtham et al., 2024). The interactions between *Dendrobium* and its microbiome not only enhance the production of secondary metabolites, which are vital for plant defense, but also contribute to the overall resilience of the plant (Pandey et al., 2023; Yu J. B. et al., 2024). These secondary metabolites can act as natural pesticides, further aiding disease suppression.

## 5 Engineered rhizosphere microbiome to enhance plant growth and stress tolerance

The rhizosphere microbiome of *Dendrobium* orchids can be engineered to enhance plant growth and stress tolerance. This engineering involves the modification of specific microbial communities, particularly plant growth-promoting rhizobacteria (PGPR), which play a crucial role in improving plant health under various stress conditions (del Carmen Orozco-Mosqueda et al., 2018). These beneficial microbes can enhance nutrient uptake, promote growth, and increase resilience to abiotic stresses such as drought and salinity (Meng et al., 2024). Rhizosphere is a dynamic environment in which plant roots interact with a diverse array of microorganisms. By utilizing advanced techniques such as metagenomics and transcriptome sequencing, researchers can gain insights into the complex interactions within the rhizosphere microbiome (Meng et al., 2024; Laffon et al., 2024). This understanding allows for targeted engineering of microbial communities to optimize their beneficial effects on *Dendrobium* orchids. For instance, genetically engineered rhizobacteria can be developed to possess traits that specifically enhance plant growth and stress tolerance, thereby addressing the challenges faced by these orchids in their natural habitats (del Carmen Orozco-Mosqueda et al., 2018; Jain et al., 2021). Moreover, the role of root exudates, organic compounds released by plant roots, cannot be overlooked. These exudates serve as nutrients and signaling molecules for rhizosphere microorganisms, influencing microbial community assembly and function (Yu J. B. et al., 2024; Adeleke et al., 2022; Wang G. et al., 2022). By manipulating the composition of the root exudates, it may be possible to selectively recruit beneficial microbes that enhance the overall health and productivity of *Dendrobium* orchids. In addition, the symbiotic relationships between *Dendrobium* orchids and mycorrhizal fungi contribute to plant health. These fungi assist in nutrient absorption and can be integrated into the engineered microbiome to bolster the growth and stress resilience of orchids (Shelake et al., 2019; Grabka et al., 2022). Engineering the rhizosphere microbiome of *Dendrobium* orchids is a promising strategy to enhance their growth and stress tolerance. By leveraging the beneficial properties of PGPR, optimizing root exudate profiles, and incorporating mycorrhizal fungi, it is possible to create a robust microbial community that supports the health and productivity of these valuable plants (Singh et al., 2023; Ayaz et al., 2023; Martínez-Chávez et al., 2024). This integrated approach not only addresses the immediate challenges faced by *Dendrobium* orchids but also contributes to sustainable agricultural practices.

## 6 Microbiome dynamics and their mechanisms to shape their rhizosphere environment

**Figure 2:**
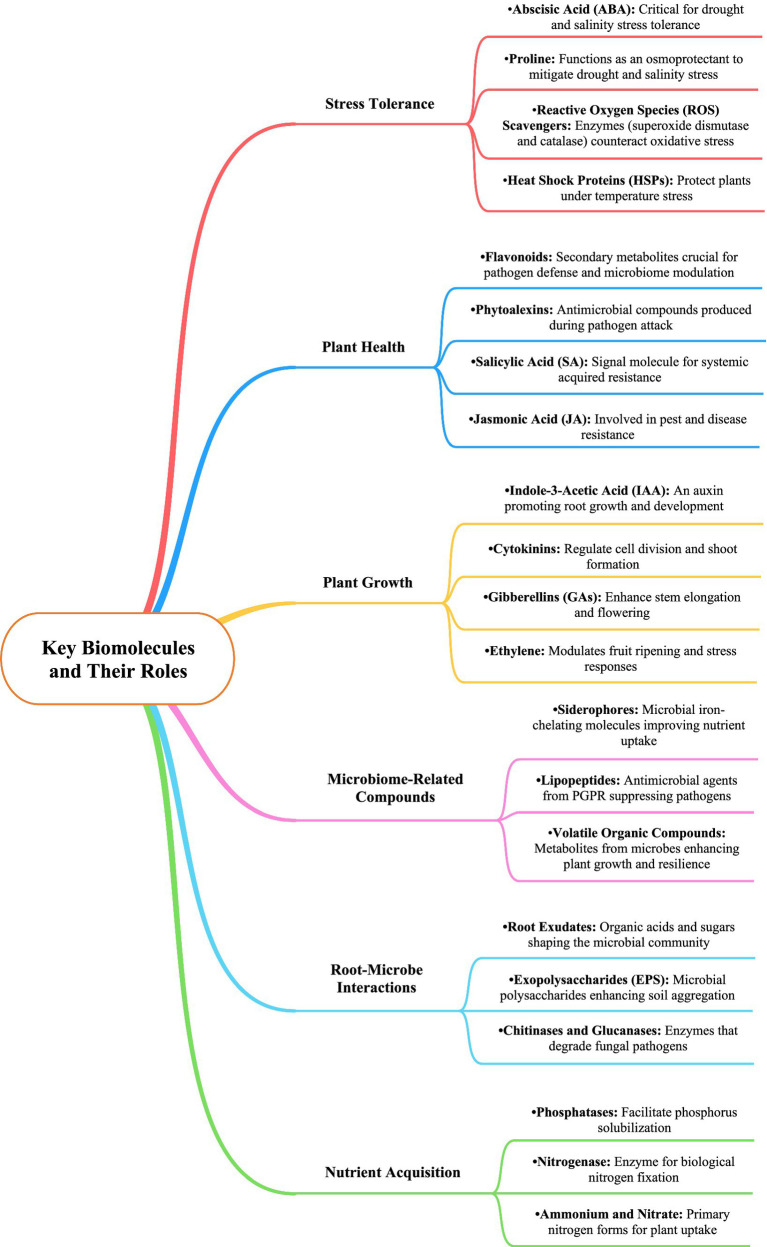
Key biomolecules and their roles in *Dendrobium* plant stress tolerance, health, growth, microbiome interactions, and nutrient acquisition.

**Figure 3:**
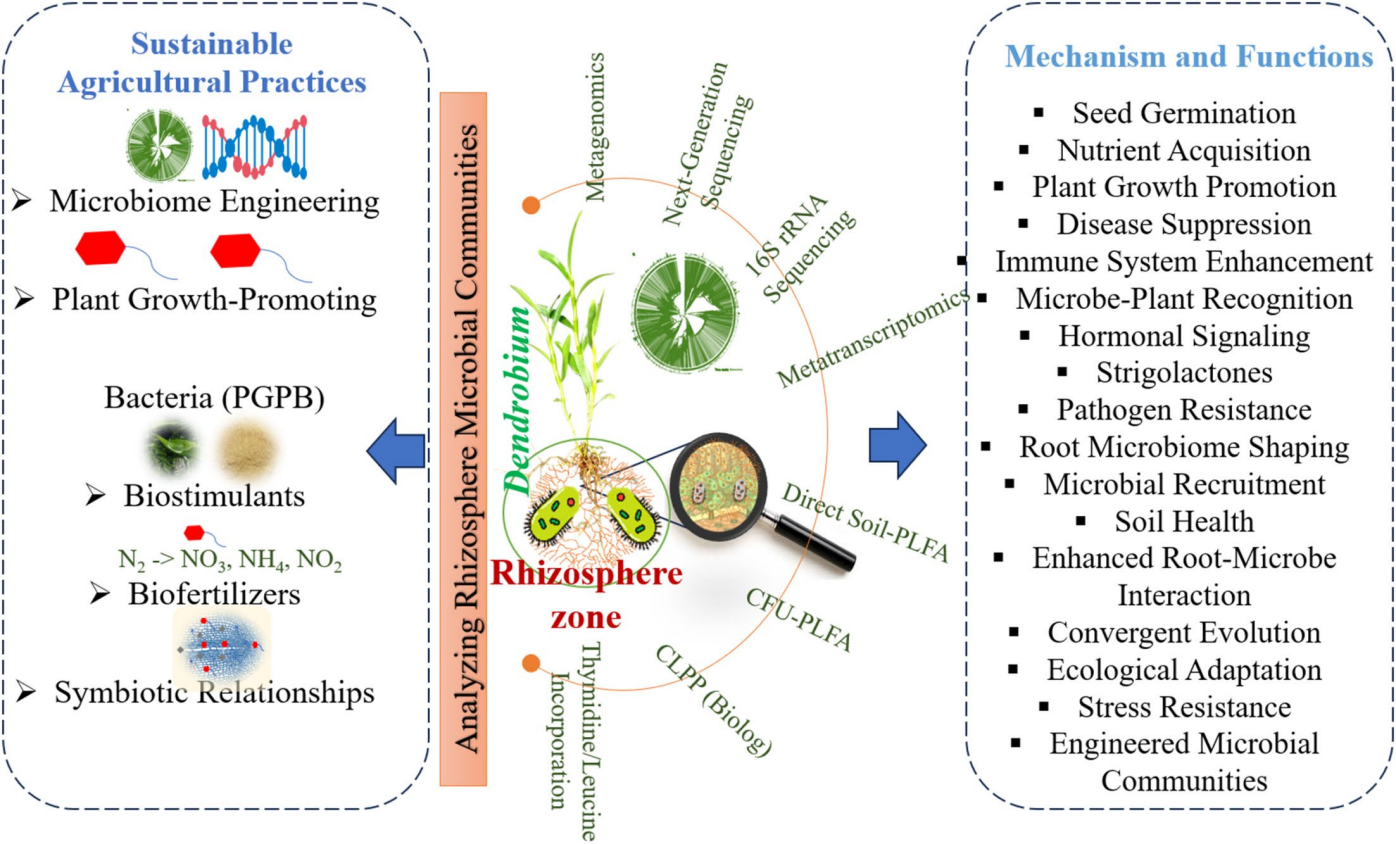
Sustainable practices and microbial mechanisms for enhancing *Dendrobium* growth, health, and resilience.

Microbiome dynamics play a crucial role in the growth and development of *Dendrobium* orchids, particularly through interactions with various microbial communities in different ecological niches (Table 3 and Figures 2, 3). These interactions significantly influenced seed germination, nutrient acquisition, and overall plant health. *Dendrobium* orchids, known for their ecological adaptability, rely on symbiotic relationships with microorganisms, including plant growth-promoting bacteria and mycorrhizal fungi, to thrive in diverse environments (Xie et al., 2024; Saikia and Thakur, 2024). The growth and development of *Dendrobium* orchids are intricately linked to microbiome dynamics, which facilitate essential processes, such as seed germination and nutrient acquisition. The interplay between these orchids and their microbial partners not only enhances their adaptability to various ecological niches but also provides insights into sustainable cultivation practices. Understanding these relationships is crucial for optimizing the growth of *Dendrobium* orchids in both natural and artificial environments (Sarsaiya et al., 2024; Tiwari et al., 2024).

**Table 3:** Interactions and mechanisms between *Dendrobium* orchids and rhizosphere microbial communities.

Interaction aspects	Target molecules	Mechanism and functions	References
Microbiome dynamics and rhizosphere environment	Microbial communities, plant growth-promoting bacteria (PGPB), mycorrhizal fungi, flavonoids, phytohormones, strigolactones	Seed germination: support seed germination and nutrient acquisition in *Dendrobium* orchids Nutrient acquisition: assist in nitrogen and phosphorus uptake Plant growth promotion: enhance growth by providing growth regulators and metabolites	Xie et al. (2024), Saikia and Thakur (2024), Sarsaiya et al. (2024), and Tiwari et al. (2024)
*Dendrobium* functional microbiome in root environment	Flavonoids, phytohormones, phenolics, lignin, jasmonic acid (JA), salicylic acid (SA), strigolactones	Disease suppression: activation of induced systemic resistance (ISR) through plant-microbe interactions Immune system enhancement: trigger immune responses, production of phenolic compounds and lignin Microbe-mediated defense: JA and SA pathways enhance resistance to pathogens	Saikia and Thakur (2024), Singh et al. (2023), Ji et al. (2024), Chen X. L. et al. (2022), Wu et al. (2022), and Ye et al. (2019)
Key signaling molecules in plant-microbe communication	Flavonoids, phytohormones (JA, SA), lipo-chitooligosaccharides, lectins, strigolactones, amino acids, fatty acids, volatiles	Microbe-plant recognition: lipo-chitooligosaccharides and lectins mediate specific microbial interactions Hormonal signaling: JA and SA modulate defense and microbial recruitment Strigolactones: promote interactions with mycorrhizal fungi, enhancing nutrient uptake	Ji et al. (2024), Favre-Godal et al. (2020), Wu et al. (2023), and Wang et al. (2024)
Defense signaling and root microbiome composition	Jasmonic acid (JA), salicylic acid (SA), flavonoids, phenolics, lignin	Pathogen resistance: JA and SA signaling pathways modulate root microbiome to enhance resistance to pathogens like *Botrytis cinerea* Microbial recruitment: flavonoid production supports the growth of beneficial microbes such as Actinobacteria Root microbiome shaping: defense signaling alters microbial composition to enhance plant health and resilience	Chen X. L. et al. (2022), Favre-Godal et al. (2020), and Wang et al. (2021)
Recruitment of beneficial microbes via root secretions	Polysaccharides (O-acetyl-glucomannan), monosaccharides (mannose, glucose), acetyl groups	Microbial recruitment: polysaccharides secreted by *Dendrobium* roots recruit beneficial microbes, enhancing their solubility and activity Soil health: promotes beneficial microbial diversity Enhanced root-microbe interaction: acetylation facilitates interaction with microbial communities, boosting growth and soil fertility	Zhu et al. (2024), He et al. (2017), Pham et al. (2023), and Zhang et al. (2020)
Genotype-microbiome interactions and convergent evolution	Microbial communities; plant growth regulators	Convergent evolution: genotypic and environmental pressures shape microbiome composition for enhanced plant resilience Ecological adaptation: *Dendrobium* species adapt to environmental stresses through microbiome selection Microbiome-associated phenotypes (MAPs): microbiome contributes to adaptive traits such as nutrient uptake and drought resistance	Herrera et al. (2022), Tiwari et al. (2024), Seem et al. (2024), Tang et al. (2020), Qi et al. (2020), and Pandita and Pandita (2023)
Synergistic response of *Dendrobium* and microbiome	Plant growth-promoting bacteria and endophytic microbes, bioactive compounds	Stress resistance: microbiomes enhance resilience against biotic and abiotic stresses, such as drought or heat Growth enhancement: microbial consortia optimize nutrient uptake and metabolic processes Engineered microbial communities: SynComs can be designed to enhance growth, disease resistance, and environmental tolerance	Martínez-Chávez et al. (2024), Pandita and Pandita (2023), Xiong et al. (2024), and Etesami and Glick (2024)

### 6.1 *Dendrobium* functional microbiome role for shape root environment

The core “*Dendrobium* functional microbiome” plays a crucial role in enhancing the immune system of plants, thereby suppressing disease development. This microbiome comprises a specific community of beneficial microbes that interact synergistically with the innate immune mechanisms of plants, particularly through the activation of induced systemic resistance (ISR) and other defense strategies (Pandey et al., 2023; Saini et al., 2023). One of the primary ways the *Dendrobium* microbiome contributes to disease suppression is through the activation of ISR, a defense mechanism triggered by beneficial rhizospheric microbes (Ji et al., 2024). This process enhances the plant’s immune response against various pathogens, effectively priming the plant for future attacks (Singh et al., 2023). The influence of microbiome extends to the modulation of the antioxidant status of plants, which involves reprogramming defense-related enzymes to mitigate oxidative stress during pathogen challenges (Chen X. L. et al., 2022). This reprogramming is essential for maintaining cellular integrity and function under stress conditions, thereby fortifying plant defenses. Moreover, activation of the phenylpropanoid pathway is another critical aspect of how the *Dendrobium* microbiome enhances plant immunity. This pathway leads to the production of phenolics and lignin, which are vital for strengthening plant defenses and suppressing disease development (Saikia and Thakur, 2024; Wu et al., 2022). The accumulation of these compounds not only reinforces the physical barriers against pathogens but also contributes to the overall resilience of the plant. The interaction between the *Dendrobium* microbiome and the plant immune system is further characterized by the recognition of microbe-associated molecular patterns (MAMPs) by plant immune receptors. This recognition triggers immune responses that are crucial for establishing a robust defense against pathogens (Adeleke et al., 2022; Tyśkiewicz et al., 2022). The interplay between MAMP-triggered immunity (MTI) and ISR creates a comprehensive defense network that enhances the ability of plants to respond to a wide range of pathogens. Additionally, beneficial microbial recruitment is a process through which *Dendrobium* plants selectively attract beneficial microbes to their rhizosphere, further enhancing their health and resistance to diseases (Mashabela et al., 2022). This selective recruitment not only improves the microbial diversity in the rhizosphere but also optimizes the interactions that bolster the immune responses of plants.

### 6.2 Key signaling molecules to facilitate plant-microbe communication

The interaction between *Dendrobium* plants and their associated microbes is mediated by a variety of key signaling molecules that facilitate communication and influence plant growth and development. Among these, flavonoids and phytohormones are crucial as they act as signaling molecules that mediate *Dendrobium* plant responses to microbial signals, thereby affecting overall plant health and productivity (Wang Y. et al., 2022). Additionally, lipo-chitooligosaccharides play a significant role in the recognition and interaction between *Dendrobium* plants and their microbial partners, enhancing the specificity of these interactions (Favre-Godal et al., 2020). Lectins, which are carbohydrate-binding proteins, also contribute to this communication by facilitating the recognition processes between plants and microbes (Siripipatthana et al., 2015). Furthermore, nodulins are involved in the signaling processes that occur during these interactions, indicating their importance in the establishment of beneficial relationships (Chen J. et al., 2022). Amino acids and fatty acids are other important signaling molecules that mediate interactions between *Dendrobium* plants and their associated microbes. These molecules can influence various physiological responses, thereby impacting plant growth and development (Wu et al., 2023). Volatiles, which are also part of the signaling repertoire, can affect plant-microbe interactions and modulate *Dendrobium* plant responses to environmental stimuli (Pandey et al., 2023). Strigolactones are particularly noteworthy in the context of symbiotic relationships. These small signaling molecules are synthesized in *Dendrobium* plants and play a crucial role in promoting interactions with beneficial mycorrhizal fungi (Wang et al., 2024). The communication between *Dendrobium* and arbuscular mycorrhizal (AM) fungi relies heavily on the exchange of strigolactones, which facilitate mutual recognition and the orchestration of complex symbiotic programs (Favre-Godal et al., 2020). The receptor D14 binds to strigolactones, activating a signaling cascade that regulates plant growth and development in response to these microbial interactions. For instance, while JA may enhance resistance to certain pathogens, SA is often associated with resistance against biotrophic pathogens, leading to a complex balance in defense strategies (Yang et al., 2022). Moreover, induced systemic resistance (ISR) activated by beneficial microbes in the rhizosphere can also involve both JA and SA signaling pathways (Ji et al., 2024). ISR is primarily mediated by JA and ethylene, suggesting that the presence of beneficial microbes can prime *Dendrobium* plants for enhanced defense against pathogens. Additionally, the production of strigolactones, which facilitate communication between plants and microbes, plays a role in influencing root development and symbiotic relationships, further enhancing the resilience of plants (Wang et al., 2018). The signaling mechanisms of *Dendrobium* are intricately linked to its microbial responses. The induction of virulence genes by coniferyl alcohol, modulation of ethylene signaling, activation of ISR, and a holistic view of the holobiont concept collectively illustrate the dynamic interplay between *Dendrobium* and its microbial partners. This relationship not only enhances the plant’s growth and stress resilience but also underscores the importance of understanding plant-microbe interactions in agricultural practices and ecological studies (Rohwer and Erwin, 2008).

### 6.3 The impact of defense signaling on the root microbiome in *Dendrobium*

Defense signaling significantly influences the root microbiome of *Dendrobium*, a genus of orchids valued for its medicinal properties and intricate microbial interactions. When exposed to biotic stress, *Dendrobium* activates defense pathways that not only enhance its own resilience but also alter the composition and functionality of its associated microbiota (Yuan et al., 2024). The root microbiome, comprising diverse microorganisms essential for plant health and metabolism, is shaped by these defense responses, with pathways like those mediated by jasmonic acid (JA) and salicylic acid playing pivotal roles (Zuo et al., 2021; Pandey et al., 2023). The JA and SA pathways play crucial roles in influencing microbial recruitment and enhancing pathogen resistance in plants, including *Dendrobium* species. JA signaling is particularly vital for plant defense against harmful organisms, such as necrotrophic pathogens and herbivorous insects (Li C. et al., 2021). For instance, the necrotrophic pathogen *Botrytis cinerea* and the bacterial pathogen *Pseudomonas syringae* are both effectively managed through JA-mediated responses in *Dendrobium* plants, which serves as a model for understanding these interactions (Wang et al., 2021). Moreover, beneficial microbes such as *Pseudomonas fluorescens* and *Pseudomonas simiae* WCS417r enhance systemic resistance in *Dendrobium* plants by interacting with JA pathways (Ye et al., 2019). These beneficial microbes can induce systemic resistance against pathogens and herbivores, highlighting the dual role of microbial species in promoting plant health and enhancing defense mechanisms. The interplay between JA and SA pathways is also significant, as they can cross-communicate in an antagonistic or synergistic manner, allowing plants to finely tune their immune responses (Wu et al., 2023). This dynamic regulation is essential for optimizing defense strategies against a variety of pathogens and pests, ultimately influencing microbial recruitment and plant resilience (Ayaz et al., 2023). Additionally, the flavonoid pathway, which is integral to plant defense and metabolic processes, is tightly linked to microbial community composition in *Dendrobium*. Flavonoid production supports the growth of microbes like Actinobacteria, which contribute to plant health by synthesizing secondary metabolites, particularly as the plant matures (Pavlova et al., 2017; Favre-Godal et al., 2020). This dynamic underscores a reciprocal relationship where the root microbiome not only responds to defense signaling but also actively participates in *Dendrobium*’s metabolic and defensive processes (Chen X. L. et al., 2022). The holobiont concept further encapsulates this interconnectedness, viewing *Dendrobium* and its microbiome as an ecological unit where microbes can enhance plant resistance to pathogens while shaping the composition and function of microbiome (Adeleke et al., 2022; Vandana et al., 2021). Overall, defense signaling in *Dendrobium* profoundly affects its root microbiome, fostering beneficial microbial communities that enhance resilience, growth, and health. Insights into these interactions hold potential for advancing the cultivation and medicinal applications of *Dendrobium* species.

### 6.4 Recruitment of beneficial microbes through chemical compounds secreted by *Dendrobium* roots

The molecular structures of the chemical compounds secreted by *Dendrobium* roots, particularly those that facilitate the recruitment of beneficial microbes, are primarily characterized by polysaccharides, including O-acetyl-glucomannan. This polysaccharide is a linear polymer composed of β-1,4-d-mannopyranosyl and β-1,4-d-glucopyranosyl residues, with acetyl groups attached at specific positions, enhancing its solubility and biological activity (He et al., 2017). Monosaccharide analysis of the polysaccharides from *Dendrobium* roots reveals a rich composition of mannose and glucose, which are essential for microbial recruitment (Pham et al., 2023). The specific molar ratios of these monosaccharides indicate a complex structural arrangement that supports interactions with beneficial microbes in the soil (Ibrahim et al., 2024). For instance, the polysaccharides from *Dendrobium huoshanense* have been shown to consist of glucose, arabinose, mannose, and rhamnose, further emphasizing the diversity of sugar components that contribute to their functional properties (Zhu et al., 2024; Li Z. et al., 2024). The presence of acetyl groups in polysaccharides plays a crucial role in their functionality. Acetylation not only enhances solubility but also affects the interaction of these compounds with other polymers, thereby influencing their extractability and biological activity (Zhu et al., 2022). The structural features of these polysaccharides can be elucidated using techniques such as nuclear magnetic resonance (NMR) spectroscopy, which provides insights into the degree of substitution of O-acetyl groups and the overall structural characteristics of the polysaccharides (Zhang et al., 2020). The linear structure of glucomannan, devoid of branches, further facilitates its interaction with microbial communities, enhancing the overall health of the soil and promoting plant growth (Wang et al., 2023). The chemical compounds secreted by *Dendrobium* roots, particularly polysaccharides such as O-acetyl-glucomannan, exhibit a complex molecular structure characterized by a rich composition of monosaccharides and acetyl groups. These structural features are pivotal in facilitating the recruitment of beneficial microbes, thereby enhancing soil health and plant growth (Zhang et al., 2022; Chen X. et al., 2023).

### 6.5 Genotype-microbiome interactions in *Dendrobium* species

Genotype-microbiome interactions in *Dendrobium* species can indeed exhibit convergent evolution across different environmental niches. This phenomenon is rooted in the complex interplay between a genetic makeup and its associated microbiome, which can significantly influence phenotypic traits such as growth and disease resistance (Chen X. et al., 2023; Sharma et al., 2024). As *Dendrobium* species adapt to varying environmental conditions, these interactions may lead to similar adaptive traits emerging independently in different populations, a hallmark of convergent evolution (Seem et al., 2024; Yuan et al., 2020; Tang et al., 2020). The concept of ecological adaptation is crucial here, as it describes how species become better suited to their environments through evolutionary changes in traits and behaviors (Tiwari et al., 2024; Qi et al., 2020; Takamiya et al., 2014). In the case of *Dendrobium*, the specific environmental niches they occupy, ranging from tropical to subtropical regions, can drive the selection of particular microbiomes that enhance their resilience to local stresses such as drought or nutrient scarcity. This selection pressure can lead to similar phenotypic outcomes, even among genetically distinct populations, thereby illustrating convergent evolution (Singh et al., 2023; Rai et al., 2023; Li et al., 2021b). Moreover, the holobiont concept emphasizes that the host organism and its microbiome function as a single evolutionary unit, influencing each other’s evolution and adaptation (Li et al., 2021a; Kaur and Sharma, 2021). This perspective is particularly relevant for *Dendrobium*, as the interactions between the plant and its microbiome can result in the emergence of microbiome-associated phenotypes (MAPs) that are beneficial for survival in specific environmental contexts (Herrera et al., 2022; Pandita and Pandita, 2023). For instance, different *Dendrobium* species may develop similar root structures or metabolic pathways to optimize nutrient uptake in response to similar soil conditions despite their distinct genetic backgrounds. Adaptive plasticity also plays a role in this process, as it allows organisms to modify their phenotypes in response to environmental variations (Song et al., 2022; Ketsa and Warrington, 2023). In *Dendrobium*, this plasticity can manifest in the form of altered growth patterns or stress responses, which may be influenced by the microbiome. Such adaptations can further reinforce the convergent evolution of traits across environmental niches. The interplay of genotype-microbiome interactions, ecological adaptation, and adaptive plasticity in *Dendrobium* species supports the notion of convergent evolution across diverse environmental niches. This complex relationship highlights the importance of both genetic and microbial factors in shaping plant responses to environmental challenges (Shelake et al., 2019; Xia et al., 2024).

### 6.6 Synergistic response of *Dendrobium* plants and their associated microbiomes

The synergistic response of *Dendrobium* plants and their associated microbiomes can be manipulated to enhance plant resilience in the face of changing environmental conditions. *Dendrobium* plants, which are one of the largest genera in the Orchidaceae family, exhibit complex interactions with various microorganisms, including plant growth-promoting bacteria and endophytic microbes. These interactions are crucial for optimizing plant health and resilience against both biotic and abiotic stresses (Saikia and Thakur, 2024; Li et al., 2023). Microbial communities associated with *Dendrobium* play a significant role in enhancing plant resilience. These communities can be optimized to support plant growth and stress resistance, particularly under adverse conditions such as drought or heat stress (Meng et al., 2024; Xiong et al., 2024). For instance, the manipulation of these microbial associations can lead to improved nutrient uptake and enhanced physiological responses, which are vital for the survival of *Dendrobium* in fluctuating environments (Joshi and Maiti, 2024; Basyal, 2024; Etesami and Glick, 2024). The application of synthetic microbial communities (SynComs) represents a promising strategy for engineering the beneficial traits of *Dendrobium* plants. By designing specific microbial consortia that can confer enhanced growth and stress tolerance, researchers can create a more resilient plant system (Pandita and Pandita, 2023; Pandey et al., 2022). Furthermore, the beneficial role of endophytic microbes that reside within plant tissues cannot be overlooked. These microbes contribute to plant health by enhancing growth and providing protection against pathogens and environmental stresses (Zhao R. et al., 2023; Chen X. et al., 2023). Their ability to produce bioactive compounds further supports the notion that manipulating microbial interactions can lead to significant improvements in plant resilience. The synergistic relationships between *Dendrobium* plants and their microbiomes can be strategically manipulated to enhance their resilience against environmental changes. By leveraging the beneficial properties of plant-associated microorganisms, including microbial inoculants and engineered communities, it is possible to foster a more robust and sustainable approach to orchid cultivation and conservation (Singh et al., 2023; Wang S. S. et al., 2022; Martínez-Chávez et al., 2024). This not only aids in the survival of *Dendrobium* species but also contributes to broader ecological and agricultural sustainability efforts.

## 7 Applications in sustainable cultivation of *Dendrobium*

Sustainable cultivation of *Dendrobium* orchids can significantly benefit from microbiome engineering, biostimulants, and biofertilizers, which collectively enhance plant growth and resilience (Table 4). Microbiome engineering involves the manipulation of microbial communities associated with plants to improve their growth, nutrient uptake, and overall health. This approach, termed “*in situ* microbiome engineering,” leverages host traits to select beneficial microbial communities that can enhance the fitness of *Dendrobium* plants (Misu et al., 2024). In the context of *Dendrobium* cultivation, plant growth-promoting bacteria (PGPB) plays a crucial role in *Dendrobium* cultivation. These beneficial microorganisms colonize plant roots and enhance growth through mechanisms such as nitrogen fixation and phytohormone production (Yang et al., 2014; Tsavkelova et al., 2016). The integration of PGPB into cultivation practices can lead to improved nutrient availability and stress tolerance, which are essential for the successful propagation of *Dendrobium*, especially in challenging environments (Kisvarga et al., 2022; Quambusch and Winkelmann, 2018). Biostimulants, including natural substances and microorganisms, further support plant health by enhancing nutrient uptake and promoting stress resilience. The application of biostimulants has been shown to increase root growth and nutrient acquisition, thereby improving the overall quality of *Dendrobium* plants (Kisvarga et al., 2022; Bhattacharyya et al., 2021). For instance, the use of seaweed extracts and protein hydrolysates as biostimulants can lead to significant improvements in plant metabolism and growth characteristics, making them valuable tools in sustainable agriculture (Shahrajabian and Sun, 2022; Makhaye et al., 2021). Biofertilizers, which contain living microorganisms, also contribute to the sustainable cultivation of *Dendrobium* by increasing the availability of essential nutrients such as nitrogen and phosphorus (de Medeiros et al., 2023). These microorganisms, including nitrogen-fixing bacteria and phosphate-solubilizing microbes, enhance the soil quality and promote plant growth through natural processes. The use of biofertilizers aligns with sustainable agriculture practices by reducing the need for chemical fertilizers and improving soil health (Saikia and Thakur, 2024; Herrera et al., 2022). Moreover, the symbiotic relationships between *Dendrobium* and various microorganisms can be harnessed to optimize seed germination and propagation *in vitro*. Understanding these biotic interactions is crucial for developing effective cultivation strategies that ensure the conservation and reintroduction of *Dendrobium* species (Teixeira da Silva et al., 2015a, 2015b; Tan et al., 2014).

**Table 4:** Sustainable agricultural practices through microbial interactions and technologies.

Sustainable practice	Mechanism	Benefits	References
Microbiome engineering	Manipulation of microbial communities to improve plant health and nutrient uptake	Enhanced plant fitness, growth, and stress resilience	Misu et al. (2024)
Plant growth-promoting bacteria (PGPB)	Colonization of roots by beneficial microbes enabling nitrogen fixation and phytohormone production	Improved nutrient availability and stress tolerance, especially in challenging environments	Yang et al. (2014), Tsavkelova et al. (2016), Kisvarga et al. (2022), and Quambusch and Winkelmann (2018)
Biostimulants	Application of natural substances (e.g., seaweed extracts and protein hydrolysates) and microorganisms	Increased root growth, nutrient acquisition, and plant metabolism	Sun et al. (2023), Kisvarga et al. (2022), Bhattacharyya et al. (2021), Shahrajabian and Sun (2022), and Makhaye et al. (2021)
Biofertilizers	Use of microorganisms such as nitrogen-fixing bacteria and phosphate-solubilizing microbes	Enhanced soil quality, nutrient availability, and reduced reliance on chemical fertilizers	Saikia and Thakur (2024), de Medeiros et al. (2023), and Herrera et al. (2022)
Symbiotic relationships	Leveraging microbial symbiosis to optimize *in vitro* seed germination and propagation	Conservation and reintroduction of *Dendrobium* species	Teixeira da Silva et al. (2015b) and Tan et al. (2014)

## 8 Bridging knowledge gaps and exploring emerging research areas in integrating rhizosphere microbiome research with precision agriculture

Integrating *Dendrobium* rhizosphere microbiome research with precision agriculture presents significant challenges and promising future directions. One of the primary challenges lies in the existing knowledge gaps in microbiome research, particularly regarding the specific roles and interactions of microbial communities in the rhizosphere of *Dendrobium* plants. These gaps hinder our understanding of how these microorganisms influence plant health, nutrient uptake, and disease resistance, which are critical for enhancing crop productivity and sustainability in precision agriculture (Xie et al., 2024; Chen L. et al., 2023). Emerging research areas have focused on the integration of microbiome studies with precision agriculture techniques. This interdisciplinary approach aims to leverage technological innovations to monitor and manage the complex interactions between plants and their associated microbial communities (Chen L. et al., 2023; Sarsaiya et al., 2024; Zhao M. et al., 2023). For instance, advancements in molecular biology and genomics are enabling researchers to explore the diversity of rhizosphere microorganisms that are often uncultivable in laboratory settings, thus providing deeper insights into plant-microbe interactions (Yu J. B. et al., 2024; Meng et al., 2024). Moreover, understanding the functionality of the microbiome can lead to the development of biological control agents that enhance crop resilience against pests and diseases, thereby improving the effectiveness of precision agriculture practices (Saikia and Thakur, 2024; Fite et al., 2023). However, the integration of these findings into practical applications remains a challenge because of the complexity of microbial interactions and the variability of environmental conditions that affect crop growth. Future research in this field should prioritize addressing the knowledge gaps identified in microbiome research. This includes investigating the specific mechanisms through which rhizosphere microorganisms contribute to plant health and productivity as well as their potential roles in mitigating environmental stresses (Zhu et al., 2024; de Medeiros et al., 2023). Additionally, fostering interdisciplinary research approaches that combine insights from microbiology, agronomy, and technology will be crucial in developing innovative solutions to enhance agricultural practices (Chen X. L. et al., 2022; Kaur and Sharma, 2021). While the integration of *Dendrobium* rhizosphere microbiome research with precision agriculture faces several challenges, particularly in terms of knowledge gaps and complexity of microbial interactions, the potential benefits are substantial. By focusing on emerging research areas and leveraging technological advancements, the agricultural sector can improve crop yields and sustainability, ultimately contributing to food security and environmental health (Teixeira da Silva et al., 2015b; Ayaz et al., 2023).

## 9 Conclusion

The rhizosphere microbiome plays a pivotal role in shaping the health and growth of *Dendrobium* species and contributes significantly to nutrient acquisition, disease resistance, and stress tolerance. Intricate plant-microbe interactions, driven by specific signaling molecules such as flavonoids, phytohormones, and strigolactones, not only enhance plant resilience but also foster beneficial microbial communities that facilitate ecological balance in the rhizosphere. Understanding these mechanisms opens the door for innovative approaches to improve sustainable agricultural practices, particularly in the cultivation of high-value crops such as *Dendrobium*. Advances in metagenomics, next-generation sequencing, and other cutting-edge techniques have provided deeper insights into the composition and functional diversity of the rhizosphere microbiome, enabling the design of engineered microbial consortia (SynComs) to optimize plant growth and disease resistance. Future research should focus on bridging the gaps in our understanding of genotype-microbiome interactions, particularly how plant genetic traits influence microbial recruitment and community structure. Additionally, the exploration of emerging technologies such as microbiome engineering and precision agriculture can further enhance sustainable farming practices. These advances could lead to tailored interventions to improve soil health, reduce dependency on chemical inputs, and foster climate-resilient agricultural systems. A more integrated approach that combines microbiome science with ecosystem-level management practices is crucial for achieving long-term sustainability in *Dendrobium* cultivation and other agricultural systems.
